# Protocol for a cluster randomised controlled trial on information technology-enabled nutrition intervention among urban adults in Chandigarh (India): SMART eating trial

**DOI:** 10.1080/16549716.2017.1419738

**Published:** 2018-01-26

**Authors:** Jasvir Kaur, Manmeet Kaur, Jacqui Webster, Rajesh Kumar

**Affiliations:** ^a^ Department of Community Medicine, School of Public Health, Post-graduate Institute of Medical Education and Research, Chandigarh; ^b^ Public Health Advocacy and Policy Impact, The George Institute for Global Health, The University of Sydney, Sydney, Australia

**Keywords:** Diet, fat, sugar, salt, fruit, vegetable, health promotion, nutrition behaviour, information technology, health education, non-communicable diseases, prevention

## Abstract

Nutrition is an important determinant of health. At present, nutrition programs in India mainly emphasize improving maternal and child nutrition. Adult nutrition has not received due attention, though diseases like hypertension and diabetes are largely preventable through changes in dietary and physical activity behaviour. Little is known about the best approaches to improve dietary behaviours, especially the role of modern information technology (IT) in health education. We describe the protocol of the SMART Eating (Small, Measurable and Achievable dietary changes by Reducing fat, sugar and salt consumption and Trying different fruits and vegetables) health promotion intervention. A Cluster Randomised Controlled Trial will evaluate the effect of an IT-enabled intervention on nutrition behaviour among urban adults of Chandigarh, India. Formative research using a qualitative exploratory approach was undertaken to inform the intervention. The IT-enabled intervention programme includes website development, Short Message Service (SMS), e-mail reminders and interactive help by mobile and landline phones. The IT-enabled intervention will be compared to the traditional nutrition education program of distributing pamphlets in the control group. The primary outcome will be the percentage of study participants meeting the dietary intake guidelines of the National Institute of Nutrition, Hyderabad, India and the change in intake of fat, sugar, salt, fruit and vegetables after the intervention. The difference in differences method will be used to determine the net change in dietary intakes resulting from the interventions. Measurements will be made at baseline and at 6 months post-intervention, using a food frequency questionnaire. The formative research led to the development of a comprehensive intervention, focusing on five dietary components and using multi-channel communication approach including the use of IT to target urban North Indians from diverse socio-economic backgrounds. The Cluster Randomised Controlled Trial design is suitable for evaluating the effectiveness of this IT-enabled intervention for dietary behaviour change.

## Background

Diet is one of the important social determinants of health. Over a period of time, due to industrialisation and urbanisation, diets have changed. Dietary intakes of fat, sugar, salt, or fruit and vegetable depend more on taste, culture, affordability etc. rather than on the dietary recommendations [1]. Australian adults get about 36% of their energy from energy-dense nutrient-poor foods (41% saturated fat and 47% sugar) [2]. High consumption of fat (60.7 g/day) and sugar (73.1 g/day) has been reported among Indians [3]. A mean salt intake of 12.5 g/day has been reported among 17 countries. Indians consume 10.98 g salt per day [4,5]. In parallel with high fat, sugar and salt consumption, low consumption of fruits and vegetables is common throughout the world. Only 5.6% Australians and 26% Indians meet WHO guidelines for fruits and vegetables (400 g/day) [6–8].

India is suffering from the double burden of malnutrition. More than 58.4% children, 53% women and 23% men are suffering from some form of anaemia. On the other hand, overweight and obesity increased by 8.1% in women and 9.3% in men between 2005 and 2016 [9]. Obesity is a major risk factor for chronic diseases such as cardiovascular diseases, diabetes, and certain cancers [10]. Most of these diseases can be prevented by changing diet and increasing physical activity [1].

Several interventions have been used to modify dietary and physical activity behaviours. Most of these interventions have targeted either diets or physical activity separately. Face-to-face individual counseling with an interpersonal component or group education sessions are common approaches [11]. Techniques that provide specific education tailored to individual needs have been more effective than generic nutrition education [12]. However, the high costs of such interventions make them impractical for population use [13]. As unhealthy diets are prevalent among the majority of people in most populations, nutrition interventions should reach a large number of people at low cost.

Web-based computer interventions present an alternative approach which allows individualized education in a cost-effective way [14]. Follow-up by email or phone has also shown encouraging results [15]. The use of mobile health applications continues to increase [16]. However, the effectiveness of information technology to improve dietary behaviours has not yet been established [17,18]. Since information technology tools are now widely available [19], we planned a cluster randomised controlled trial (CRCT) to test the effectiveness of an information technology-enabled nutrition education intervention called ‘SMART (Small, Measurable and Achievable dietary changes by Reducing fat, sugar and salt consumption and Trying different fruits and vegetables) Eating’. The objective of this paper is to describe the design and approach of the SMART Eating Trial.

## Methods

### Overview

**Figure 1.**
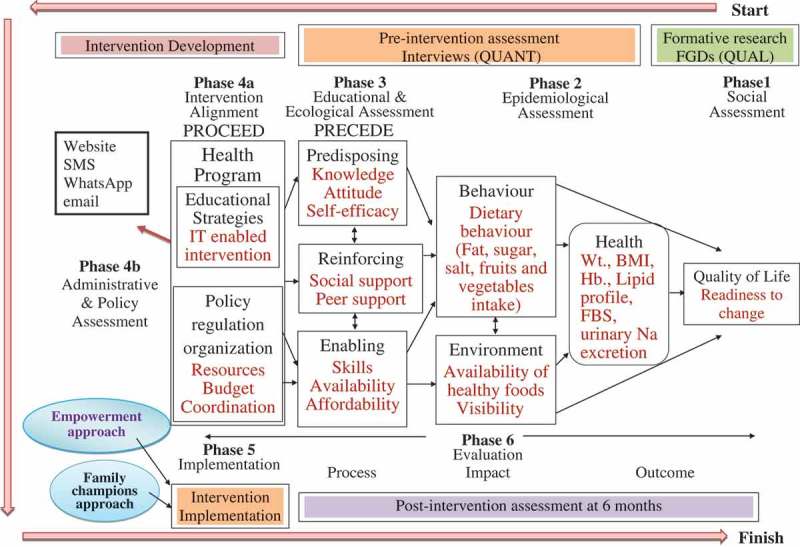
PRECEDE-PROCEED Model (Green & Kreuter) [20].

The approach is based on the PRECEDE-PROCEED Model (Figure 1) [20]. Formative research was conducted to inform the development of the intervention (Supplementary file 1). A cluster randomised controlled trial (CRCT) design was employed to determine the effectiveness of the SMART Eating Intervention on an individual as well as at family level.

### Study setting

A sector of Chandigarh city, located in northern India, has been chosen for the study. The selected sector has a population of about 30,000 and includes people of all socio-economic strata with the type of housing, assigned by the Chandigarh Administration as low-income group (LIG), middle-income group (MIG) and high-income group (HIG), taken as proxy for socio-economic status. This sector has been purposely selected because The Community Medicine Department of The Post Graduate Institute of Medical Education and Research (PGIMER), Chandigarh, has been working here to strengthen healthcare services, and use of mobile phones and internet is also widespread in this area.

About 89% households in Chandigarh use mobile or landline phones [21]. A rapid survey of the study area in 120 families from different types of housing during planning phase of the CRCT revealed that all families, including those from low-income group (LIG), had mobile phones. Most of these families had smart phones and internet services. India will have 450 to 465 million internet users in 2017 [22]. Mobile internet penetration is also high in India with 400 million internet users accessing the internet through mobile phones [23,24].

### Study design

**Figure 2.**
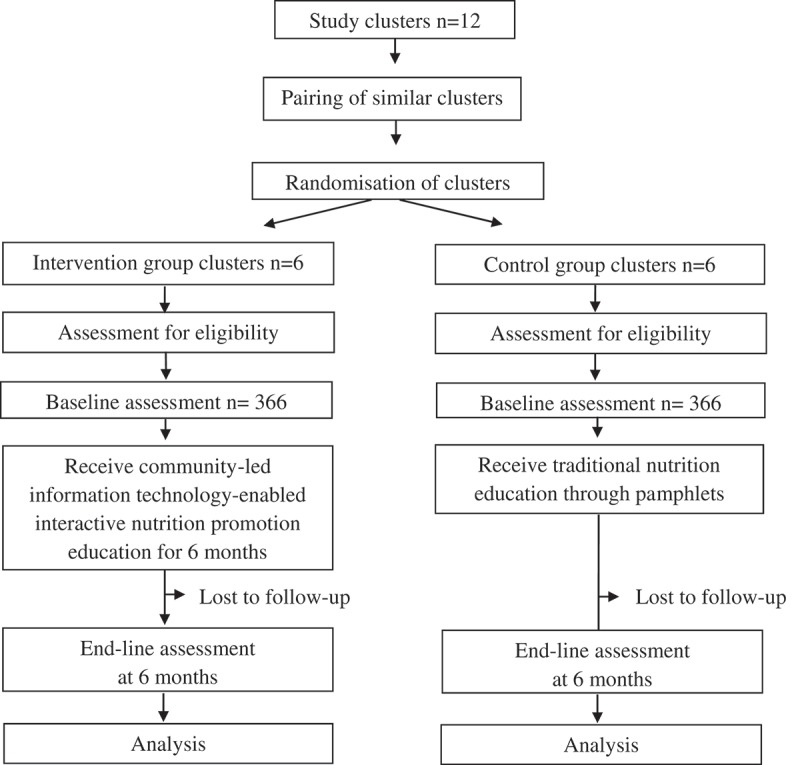
Study design.

A cluster-randomised controlled trial design with two groups will be employed: (1) The intervention group will receive community-led information technology-enabled SMART Eating Intervention. (2) The control group will receive traditional nutrition education through pamphlets (Figure 2). The objectives of the intervention are to: (a) Reduce the consumption of fat, sugar and salt by 20%; (b) Increase fruit and vegetable consumption by 20%; (c) Reduce the monthly purchase and consumption of fat, sugar and salt; (d) Increase the weekly purchase of fruits and vegetables; and (e) Identify barriers to dietary behaviour change and help families develop coping strategies.

### Sampling

A multi-level sampling strategy will be used. Twelve clusters, based on the type of housing (LIG, MIG and HIG), have been selected bearing in mind that there is sufficient geographic separation to avoid any spill-over effect of the intervention. Similar clusters will be paired together to form six pairs. For each of the six pairs, computer generated randomisation will be used to allocate clusters to intervention and control groups by a researcher not involved in the study. The number of clusters and cluster size will be fixed. Equal numbers of families will be recruited from each cluster through systematic random sampling. One adult (35–70 years) per family will be randomly selected as the index case for the baseline and post-intervention assessment.

One family champion (the adult member who cooks food most of the time) will be selected from each family for the implementation of the intervention. In recognition of the fact that not all family champions will be using information technology tools or mobile phones (as demonstrated through the formative research), one co-champion will also be selected from each family. A co-champion can be any person from the family identified by the family champion who would be able to assist him/her in using the internet and mobile phone communications.

### Inclusion and exclusion criteria

The inclusion criteria for recruitment of study participants will be: families from low, middle and high-income group housing (LIG, MIG, HIG); residing in the study area 6 months or more; having access to mobile phone/landline phone/internet; and including adults between 35 and 70 years of age. This age group has been selected because most of the youth (20–34 years) move away due to higher education and job opportunities etc. Moreover, adults between 35 and 70 years of age have higher chance of developing chronic diseases. Pregnant women and families not providing consent to participate in the study will be excluded.

### Sample size

Sample size calculations were done for each of the outcome variables: fat, salt, and fruit and vegetable intake, but not for sugar intake, because estimates of sugar intake were not available. The prevalence of adequate dietary intake for the different foods and nutrients was used to calculate the sample size for fat and fruit and vegetables based on dietary surveys and based on urinary salt excretion for salt. As no similar trials were available to inform an assumption of improvements in the intervention arm, the sample size was calculated based on a 20% improvement. Sample size for estimation of change in salt required the largest sample size. Based on a previous study, which indicated that 15% of adults had a salt intake of <5 gram per day [25], and assuming a 20% improvement in the intervention arm, power calculations (alpha = 0.80; p = 0.05) indicated that 83 participants would be needed per study arm. As the intra-cluster correlation co-efficient was not available for the outcome of interest, we used a design effect of 2, which increased the estimated sample size to 166 [26]. Taking into account a drop-out rate of 10%, we calculated that 183 participants would be required [27]. However, in order to enable comparison of outcomes between different age and socio-economic groups, the calculated sample size had to be doubled. Thus, a final sample size of 366 participants per study arm was deemed to be sufficient to assess changes in each of the study outcome.

### Primary outcome

The primary outcome for the study is the % change in study participants meeting India’s National Institute of Nutrition (NIN) dietary guidelines for fat, sugar, salt, fruit and vegetable intake. The NIN recommend that, for adults, 20–30% energy should be from fat (10% from visible fat and 10–20% from foods other than visible fat); no more than 20 g sugar for women and 25 g sugar for men, less than 5 g salt, and at least 100 g of fruits and 300 g of vegetables per day.

### Secondary outcomes

Secondary outcomes include % change in body weight, and mean change in body mass index (Kg/m^2^), blood pressure (mm Hg), haemoglobin (g/dL), fasting plasma glucose (mg/dL), total serum cholesterol (mg/dL), high-density lipoprotein (mg/dL), low-density lipoprotein (mg/dL), triglycerides (mg/dL) and urinary salt excretion (g/day).

### Intervention development

The use of theoretical models increases the likelihood for an intervention to be effective [28]. Hence, the PRECEDE-PROCEED Model (Figure 1) was used to inform the intervention [20] and the behaviour change matrix was used to guide the selection of channels and methods of communication (Table 1).

**Table 1.** Dietary behaviour change matrix.

Communication Objective	Audience	Stage of Change of the audience	Facilitating factors	Strategies*	Channels of communication	Methods of communication	Frequency of messages	Expected outcomes
To increase fruit intake	Primary Audience: Individual	Pre-contemplation	Women being main chef of the family	1. Involving family members as co-champions	1. Interpersonal communication	1. Verbal	1. SMS: weekly	1. Increase knowledge of dietary recommendations
To increase vegetable intake	(Family champion & Co-champion)		Easy availability of fruits and vegetables in the study area	2. Awareness	2. Tele-communication	2. Flip book	2. WhatsApp: weekly	2. Increase awareness about own dietary intake and benefits of SMART eating.
To reduce fat intake	Secondary Audience: Family		Accessibility to fruits and vegetables through markets and food vendors	3. Emphasis on seasonal fruits and vegetables	3. Internet	3. SMS	3. Email: weekly	3. Increase risk perception for nutrition related diseases
To reduce sugar intake				4. Visibility		4. WhatsApp: text, images & videos	4. Landline: need based	4. Increase their self-efficacy for changing dietary behaviour
To reduce salt intake				5. Good cooking practices		5. Website		5. Improve their dietary behaviour
				6. Avoidance/Substitution		6. e-mail		6. Increase the purchase of fruits and vegetables and reduce their monthly consumption of fat, sugar and salt
				7. Emphasis on sharing		7. Educational aids: Dining table mat, Kitchen calendar and pamphlets		
				8. Building self-efficacy		8. Measuring spoons (5g)		
				9. Emphasis on cutting down on medical bills				
				10. Kitchen gardening if feasible				

*Details regarding strategies are available in the text of the article

Taking into account the available literature on nutrition education interventions, the National Institute of Nutrition dietary guidelines, and findings from the formative research (Supplementary file 1), the contents of the nutrition education program were prepared [29]. This included the health benefits of eating more fruits and vegetables and reducing consumption of fat, sugar and salt; dietary guidelines, pictorial portion guides (for estimating the amount of fruits and vegetables that should be consumed in a day); the importance of using a measuring teaspoon (for estimating the use of fat/oil, sugar and salt); tips to increase fruit and vegetable intake and to reduce fat, sugar and salt intake; body mass index; frequently asked questions and a quiz on fat, sugar, salt, and fruit and vegetables. A SMART eating kit has been prepared which includes several educational aids such as a dining tablemat, kitchen calendar and measuring spoons. A SMART eating flip book has also been prepared. The IT intervention includes the SMART eating website development with an interactive help function, short message service (SMS) applications in the form of text, videos and images and e-mail. A nutrition education pamphlet has also been prepared for the control group.

All intervention tools were designed by the first author (JK). Content validation was done by eight experts from related fields in PGIMER, Chandigarh. The website design was finalised by two co-authors and one nutrition expert. The tools were pre-tested on five health professionals and 15 families from different socio-economic groups (5 families each of LIG, MIG and HIG) and modifications were made.

### Intervention components

The nutrition education intervention will have two components (Table 2). The *Interpersonal Component* includes: home visits for training of family champions and co-champions using a flip book over a period of one month; distribution of the SMART Eating kit with written information in Hindi language; and health check-up reports (lipid profile, haemoglobin and fasting plasma glucose etc.) after the baseline survey. *Information Technology (IT) Component* will be implemented, after training of family champions, through mobile phone, landline phone and internet communications.

**Table 2.** Description of intervention components.

Intervention components			Duration	Description
1	Interpersonal component			
	a	Training of family champions and co-champions	1 month	Face-to-face training will be provided using flip book on how to use different features of the intervention on internet and mobile phones or tablets.
	b	Distribution of SMART Eating kit		Kit containing innovative educational aids in the form of a dining table mat, kitchen calendar and spoons will be provided to all families.
		A dining table mat		A laminated dining table mat on dietary recommendations for fat, sugar, salt, fruits and vegetables, one side of which will guide the families on vegetables and fruits available in summer and winter season, how much and when to eat. The other side of the mat will remind family members of consuming less oil, sugar and salt by reducing the intake of foods high in fat, sugar and salt.
		Kitchen calendar		A pictorial kitchen calendar for display in the kitchen to remind family members of using less fat, sugar and salt while cooking.
		Measuring spoons		A set of three measuring spoons will be provided to help families measure the amount of fat, sugar and salt they use.
	c	Tailored nutrition education		Nutrition education will be tailored to the specific needs of family members e.g. those already suffering from diabetes and high blood pressure and those at high risk of developing these chronic conditions such as family history of diabetes, hypertension and obesity etc.
	d	Health check-up		Blood test reports of lipid profile, haemoglobin and plasma glucose will be delivered at their door steps for motivating them to modify their dietary behaviour.
2	Information technology component			
	a	Tele-communication		
		SMS	6 months	SMS will be sent weekly in Hindi language
		WhatsApp		WhatsApp messages will be sent weekly in English, Hindi and Punjabi.
		Landline		Landline phone will be used to take feedback from family members if anyone do not reply to mobile phone call
	b	Internet		
		SMART Eating website	6 months	SMART Eating website available in three languages (English, Hindi and Punjabi) will be password protected to prevent spill-over effect. Each family will be provided with individual password to login. Password will be sent through SMS, WhatsApp and email.
		Content delivery		Content will be delivered in parts. New content will be added every fortnightly.
		Using content on website		Content will be used by the participants on their own
		Query box for interactive help		Interactive help will be provided through query box or e-mail for any time assistance to discuss any difficulties in using the intervention or adherence to dietary changes. Through this participants can drop their questions in the query box at any time and responses will be given by the experts in the field of nutrition. In addition, query box will have provision for discussion among the participants.
		● Quiz and frequently asked questions (FAQs)		Quiz on fat, sugar, salt, vegetables and fruits and FAQs

### Intervention implementation

The intervention will be implemented at a family level using the family champion approach – an adaptation of health champion approach [30] – and the ‘Empowerment approach’ to build capacity of the individuals and families [31]. The family champion approach will motivate all other family members to eat less fat, sugar and salt and consume more fruit and vegetables. The multi-channel communication approach includes interpersonal communication, tele-communication (mobile phone) and internet (website). The same intervention will be delivered to all intervention families. Clear instructions for families not to share intervention messages with other families or friends in the study area will be given.

Intervention implementation strategies will include:

- *Involvement of family members*: especially children as co-champions with a view to increasing self-efficacy for dietary changes.
- *Creating awareness*: by providing information on nutrition.
- *An emphasis on seasonal fruits and vegetables*: which are less expensive at their peak season, and are full of nutrients.
- *Increasing visibility*: by placing a tray containing fruits and vegetables on the dining room or kitchen table, and keeping snacks and food items high in fat, sugar and salt in places less visible to family members.
- *Highlighting good cooking practices*: such as methods requiring less fat, sugar and salt.
- *Avoidance and substitution*: avoiding food too high in fat, sugar and salt or substituting snacks with fruits and vegetables.
- *Emphasis on eating by sharing*: fruits and vegetables among family members whatever the amount available.
- *Building self-efficacy*: SMART eating kit articles to increase the self-efficacy in changing dietary behaviour.
- *Cutting down on the medical bills*: emphasising the benefits of increasing fruits and vegetables and decreasing fat, sugar and salt to prevent chronic diseases which in turn may help to reduce medical bills.
- *Kitchen gardening*: Encouraging, if feasible, kitchen gardening or growing vegetables in earthen pots.

### Intervention in the control group

Control group families will be provided with a pictorial pamphlet on dietary recommendations from the National Institute of Nutrition, India. The content and pictures in the pamphlet will be the same as that used for the dining table mat to be provided to intervention group families. One side will have information on seasonal fruits and vegetables along with dietary recommendations; the other side will have pictures of measuring spoons showing the amount of salt, sugar and fat in one spoon; dietary recommendations; pictures of foods high in fat, sugar and salt; and information on reducing the intake of these nutrients. The pamphlet will be in Hindi and will be provided to study participants along with their blood test reports. Participants will be asked to read the information provided in the pamphlet in their own time, to make changes to their diet accordingly and to convey the same information to their family members.

### Data collection

The following tools have been developed for data collection: (1) Household profile proforma to identify Index case; (2) Structured Questionnaire for Index case: Part A – Socio-demographic data, medical history and physical measurements, Part B – ‘Stages of change’ questions based on those developed by Lechner *et al* (1998) to assess participant’s self-rated dietary intake [32] and Part C – Attitude, social influence and self-efficacy questions based on ‘Attitude-Social influence-(self)-efficacy model’ (ASE model) using a five point Likert scale [33]; (3) Proforma for selecting family champion and co-champion; (4) Information technology use proforma; (5) A validated Food Frequency Questionnaire (FFQ) for individual dietary assessment [34]; (6) Household food purchase and consumption record proforma to assess food consumption at household level [34]; (7) Sphygmomanometer, weighing scale, anthropometric rod, and laboratory equipment for collecting blood and urine samples; and (8) Feedback proforma.

Height will be measured to the nearest 0.1 cm using the anthropometric rod. Weight will be measured with minimum clothing without shoes to the nearest 0.1 kg on a portable electronic weighing scale. Blood pressure will be measured twice using a standard instrument to the nearest 2 mm Hg [35]. Blood samples (5 mL: 1 mL-EDTA vial, 1 mL-Oxalate vial and 3 mL-plain vial) will be obtained to estimate haemoglobin, plasma glucose (mg/dL), total serum cholesterol (mg/dL), high-density lipoprotein (mg/dL), low-density lipoprotein (mg/dL) and triglycerides (mg/dL) using standard laboratory methods. Instructions regarding overnight (12 hrs.) fasting will be given to all participants one day prior to blood sample collection [36]. Morning fasting urine samples (5 mL) will be collected in urine containers. Participants with blood pressure above 140/90 mm Hg in both intervention and control groups and those with abnormal blood test results will be referred to doctors in the local health centre.

After implementing the intervention for a period of one month, all participants will be invited, through a SMS, phone call or home visit, to ask whether they have made some changes to their diets. For process evaluation, feedback will also be taken from the participants using a proforma. A log book will be maintained to record barriers and enablers related to the use of the IT-enabled intervention as well as identifying actions that could be taken to improve its use. Qualitative in-depth interviews using ‘Extreme or Deviant case’ sampling will also be conducted to understand barriers and facilitators.

A team of three members, the first author and two other members who are outsiders to the community, will collect the data. The team members have received training on data collection. Neither the investigator nor the data collectors will be blind to intervention. Intervention implementation, process evaluation, and quality control will be conducted by the first author.

### Data analysis

The quantitative data analysis will be performed using Statistical Package for Social Sciences (SPSS) version 21 based on Intention-to-treat analysis. The cluster design will be taken into consideration during analysis and cluster effect will be reported. Descriptive statistical analysis will include calculation of sample means, standard deviation (SD) and proportions according to the type of variables. Categorical variables will be compared using chi-square test. Within group changes in quantitative variables from baseline to endline at six months will be analysed using a paired *t*-test. Unpaired *t*-tests will be applied to independent samples for between group comparisons. In order to explore potential differences between groups (e.g. socio-economic, age groups and gender etc.) multivariable regression analysis will also be used.

The FFQ data will be entered into the spreadsheet software used in the PURE study [34]. This software determines the amount of food (from the food list) consumed by the individual, by multiplying the average consumption with frequency of intake and portion size of the food item. The intake of each food item will then be re-computed for a day, and then multiplied with the nutrient amount per gram to obtain the daily nutrient intake from each food. The estimated nutrient intakes per day will then be summed to obtain the total daily nutrient intakes.

Participants’ nutrient intakes per day will be compared against the NIN dietary guidelines to calculate the percentage of participants meeting the guidelines for fat, sugar, salt, fruit and vegetable intake. Sub-group analysis by disease status will also be performed. Changes in fat, sugar, salt, fruit and vegetable intake in both the groups will be calculated as post-intervention minus pre-intervention nutrient intakes. The net intervention effect will be calculated as the change in dietary intake in the intervention arm minus the change in dietary intake in the control arm from baseline to the study end at six months (difference in differences method) [37]. Dietary information collected through food purchase and consumption records will also be used to calculate the change in per capita monthly consumption of fat, sugar, salt and fruit and vegetables for each family. The clinical significance of changes achieved will be estimated based on association of nutrient intakes with clinical outcomes reported in existing literature.

BMI will be calculated as weight (Kg) divided by height (m)^2^. Blood samples for haemoglobin, fasting plasma glucose and lipid profile, and urine samples will be analysed using standard laboratory methods. The 24 h urinary sodium excretion (mg/day) will be estimated from a single morning fasting urine sample using the Kawasaki formula [38].

Participants will be classified into five stages of change separately for fat, sugar, salt, fruit and vegetable intake: Pre-contemplation, Contemplation, Preparation, Action and Maintenance stage on the basis of quantitative data. Stage will also be assessed based on qualitative data in relation to people’s knowledge of dietary intake guidelines and their perception and awareness about their own dietary intakes and dietary practices.

The use of specific components of the intervention, satisfaction with the program and ideas for improvement will be analyzed by summarising the answers to open ended questions from the feedback proforma. Website use will be measured through feedback regarding login by number of participants and the visitor count.

## Discussion

The use of information technology is increasing and mobile health has the potential to reach large numbers of people quickly at low cost [16]. Therefore, this CRCT is planned to robustly determine the effect of this IT-enabled health promotion intervention on nutrition behaviour. The cluster RCT was chosen over an individual RCT to minimise the threat of any contamination of the nutrition intervention in an individual RCT [39]. Study clusters will be selected based on housing type because it is easier to stratify population based on the type of housing and this is a good proxy for socio-economic status in this area.

The review of literature on nutrition education interventions revealed many gaps. Most previous studies have targeted individuals at high risk of disease as ‘at risk’ individuals are more motivated than the general population; other studies have had a small sample size or non-representative sample [40–43]. Instead of overall health promotion, most of the studies to date have focused on single outcome such as reducing weight, blood pressure or cancer prevention [40,44,45]. Some trials have provided incentives to the participants in the form of provision of diets free of cost, while others made fruits and vegetables available at low cost in the study area [44,45]. In some trials, the control group did not receive any intervention which raises questions about study validity [41,46,47]. The majority of the identified interventions had no theoretical basis, and most of them used traditional methods of education such as individual/group counseling or printed material, and most did not achieve a significant change in dietary behaviour [40–45,48]. Whilst a few studies have reported change in one or another dietary behaviour using information technology tools most had one of the methodological flaws mentioned above [40,49,50,51]. Moreover, most interventions to date have been developed and evaluated in developed countries, except a few previous studies in India [49–51]. The effectiveness of interventions based on information technology has not been established as yet. This study is planned to address the above mentioned gaps.

The existing literature shows that the diseases linked to high fat, sugar and salt intake, such as heart disease, diabetes, some cancers, and nutritional deficiencies, are preventable through increased consumption of fruits and vegetables [52–59]. Yet, many studies target one or two aspects of diet, few studies target multi-components of diet. The study conducted in rural South India [60] is an exception but also focused mainly on fruit and vegetable components; with fat, sugar and salt as the secondary outcome measures. The objective of the intervention here was therefore to focus on all the five dietary components together to develop a comprehensive health promotion intervention using IT.

### Methodological considerations

Considering the potential risk of non-use of IT components by the families, process evaluation will be undertaken to assess the extent of various intervention components and identify factors hindering the use of IT-enabled intervention. In case participants do not make sufficient use of IT components, we may plan to continue the intervention by distributing printed materials.

That said, the ability to measure any changes in dietary intake will require robust assessment measures. FFQs have emerged as a useful tool in epidemiological studies across the world, and these have become popular in Indian settings because they impose fewer burden on the subjects compared to other dietary assessment methods. Our FFQ has been developed and validated in a northern Indian setting [34]. The dietary intake of the index case was considered to be representative of the family. At family level, fat, sugar, salt, and fruit and vegetable intake will also be assessed through self-reported purchase which can be used to triangulate findings from FFQ.

Assessment of salt intake from FFQs usually underestimates the intake so urinary sodium assessment provides better information [61]. Though 24-hour urinary sodium excretion is gold standard for estimating salt intake [62], there is some evidence to show that spot urine samples can be used to estimate mean change in population salt intake, and, as this poses much less of a burden to participants, and the equations to estimate 24 hour salt from spot urine samples have been previously tested in different Indian populations; our assessment is based on single spot urine samples [63]

### Contextual factors influencing study design

The existing pattern of housing (LIG, MIG and HIG), as assigned by Chandigarh administration, was taken as proxy for socio-economic status (SES). In order to ensure equal representation of all socio-economic groups in both study arms, we stratified the sample according to type of housing, and matching of clusters was done before random allocation of similar clusters in intervention and control group.

In contrast to a recent study from South India [60], our formative research indicated that participants from all SES perceived themselves at greater risk of nutrition-related diseases and even those from LIG were supportive of dietary change intervention. Therefore, we will also include LIG in the study despite the fact that affordability could be one of the barriers to SMART eating in this group [64]. In recognition of this, one of the intervention strategies will be to emphasise that money spent on junk foods which are often high in fat, sugar and salt could instead be utilised for purchasing vegetables and fruits.

### Contextual factors influencing the intervention

Our formative research found multiple opportunities for change at individual as well as family level (Supplementary file 1). It established that the majority of the target audience was at the pre-contemplation stage of change, which was different to other studies where participants were classified into various stages [65]. In contrast to developed countries where processed foods form the bulk of diet, the formative research confirmed that Indians have preference for home cooked food which is consumed by all family members. This informed our decision to implement the intervention at the family, on contrast to other studies that have intervened at the individual level. To the best of our knowledge this ‘family champion’ approach had not been used in previous nutrition interventions.

The use of IT tools, such as mobile phone and the internet, for delivering nutritional messages is considered acceptable for the target population as these tools are available in most families. In addition to IT tools, participants (in the formative research) also indicated the need for face-to-face education and provisioning of printed material which can be displayed in the house. This led to the development of the SMART Eating kit containing a kitchen calendar and dining table mat etc. In view of the fact that people lack skills in measuring the amount of fat, sugar and salt recommended, measuring spoons have been added to the SMART Eating kit.

The formative research showed that men and women were equally supportive of dietary behaviour change, but the majority of them identified women as the main facilitator for dietary behaviour change in the family as women are usually responsible for cooking the food. Easy availability of vegetables and fruits in local markets and vendors was considered to be another facilitating factor for the intervention.

### Strengths and limitations

The main strength of this study is inclusion of all socio-economic groups in a sufficiently large representative sample with focus on all dietary components. Inclusion of all socio-economic groups will likely enhance the generalisability. The calculated sample size is large compared to other RCTs which should provide strong power. Strict randomisation throughout the sampling procedure will also help to minimise any potential bias although the lack of blinding will mean the results will have to be interpreted with caution.

Involvement of stakeholders in the formative research for intervention development is another strength of our study which draws on other studies [66]. The multi-channel communication approach for delivering the IT-enabled nutrition intervention is unique. Visitor count will be used as an indicator of use of website content by the participants, however, due to lack of resources we are not able to measure the time spent on the website. This study will provide important information on the feasibility, acceptability and effectiveness of multi-strategy intervention using information technology in bringing about dietary behaviour change in a developing country but it will not provide the comparative effectiveness of each component of the intervention.

Assessment of behaviour change is proposed at 6 months after the intervention. Ideally assessment should also be done 6 months after the active intervention to assess the maintenance of behaviours. However, due to resource constraints, we may not be able to continue beyond 6 months. Another limitation of our study is the selection of only one member from each family to measure dietary changes, as it is impractical to measure dietary intake of all members of the family. This may underestimate the effect size.

Access and use of IT by family champion can be a challenge, but a rapid survey of 120 families from different types of housing in the study area suggested high prevalence of IT use. Each family had at-least one mobile phone and majority had smart phones or computers with access to internet including those living in LIG housing. It is, therefore, assumed that this intervention which has made use of available IT tools will not pose additional burden on the families.

Though cost-effectiveness has not been considered as one of the primary objective of the study, the capital and recurring cost for 5 years is estimated to be INR 889,000 for intervention group and INR 6000 for the control group. A formal cost-effectiveness analysis can also be attempted. If found to be cost-effective, this trial may pave the way for carrying out dietary behaviour change intervention on large scale so as to have a larger impact on prevention of nutrition related diseases.

## Supplementary Material

Supplementary material

## Paper context

Mostly dietary intervention studies have been conducted in developed countries; studies from developing countries are few. Effectiveness of information technology interventions on nutrition behaviour has not been tested yet in developing world. Therefore, a formative research led, comprehensive intervention, focusing on five dietary components, using multi-channel communication including IT for urban North Indians from diverse socio-economic backgrounds has been developed. The Cluster Randomised Controlled Trial can evaluate the effectiveness of IT-enabled intervention for dietary behaviour change in developing countries.
