# Supplementary material for: Protocol for a cluster randomised controlled trial on information technology-enabled nutrition intervention among urban adults in Chandigarh (India): SMART eating trial
Source: Glob Health Action. 2018 Jan 26;11(1):1419738. doi: 10.1080/16549716.2017.1419738 (PMC5795704; doi:10.1080/16549716.2017.1419738)
Supplement: Supplementary material [file ZGHA_A_1419738_SM8000.docx]

**Supplementary file 1**

**Development of an Information Technology-enabled Health Promotion Intervention for Dietary change: Formative Research**

Considering formative research as a key to understanding the dietary behaviour of our study participants and for developing the intervention, we used qualitative research approach. The purpose was to (a) understand level of the knowledge, attitudes, and practices related to nutrition, (b) identify barriers to dietary behaviour change, (c) identify the content, messages, and channels and methods of communication for developing a culturally acceptable health promotion intervention, and (d) determine the feasibility and acceptability of the planned intervention.

***Research design***

A qualitative exploratory design was used, wherein, 10 focus group discussions (FGDs) were held with the residents of the study area. We used purposive sampling and snowball technique to recruit eligible participants (above 35 years of age). The study participants were recruited with the help of President of Resident’s Welfare Association, Auxiliary Nurse Midwives (ANMs) and anganwadi workers. We decided to hold FGDs to ensure that two age groups of 35-60 years & above 60 years, both men and women, and the three socio-economic groups are covered. Therefore, 4 FGDs were held in lower socio-economic group and 4 were held in middle socio-economic group. However, in upper socio-economic group only 2 FGDs were held; one common with both men and women and one with women only. Focus group discussions were conducted till data saturation was achieved i.e., when additional new information stopped emerging.

***Data collection***

A total of 53 participants participated; about 5-7 in each of the 10 FGDs. The duration of FGDs ranged from 30 minutes to one hour. A semi-structured FGD guide was used to explore knowledge, awareness, perceptions, beliefs, barriers and facilitators for dietary behaviour change. Detailed notes were taken which were reviewed daily by the researcher. Memos for analysis were written every day to avoid any gaps in data while doing analyses.

***Data analysis***

Narrative thematic analysis, informed by framework analyses approach, and techniques from grounded theory were used for analyses. At the outset, existing models of behaviour change from literature search were used to identify ‘*a priori’* codes and data were coded against these codes. The data which did not fit into ‘*a priori’* codes was explored using thematic analysis to identify emergent codes. During data reduction, common codes were clustered together under higher concepts/categories. Then data were interpreted to understand relationships between higher concepts and their common codes.

***Findings***

We found that the structural, sociocultural and contextual conditions influence behaviours both at individual and family level. Since these are the attributes of behaviour change, therefore these sub-themes were labeled as attributes (Figure 3). Using these themes and subthemes, strategies that can influence perceived barriers among study participants for change in their diets were developed (Table 1 of design and methodology paper).

Knowledge of dietary recommendations and awareness about own dietary intake would influence the attitude towards dietary behaviour change and would raise self-efficacy. The facilitators that can help in dietary change were useful in deciding on who in the family can influence dietary change e.g., often the women are the chef in the family so should be considered the primary audience. Therefore, anyone in the family who cooks food was considered as champion. It also helped us in understanding the availability and affordability issues of families, especially for vegetables and fruits. Both fruits and vegetables were available, though, were affordable only in a specific season.

Participants in our study were at pre-contemplation stage of change. However, by the end of focus group discussions, they expressed the need for awareness on dietary recommendations and healthy food options which were experienced across all FGDs. The change strategies suggested by the FGD members were gradual change rather than changes in one go especially to change the tastes for salt and sugar, healthy cooking methods e.g., using non-stick for shallow fry, making healthy choices while eating out, avoidance of adding extra salt, sugar and oil etc. Participants indicated preference for the use of mobiles phone and internet to deliver nutrition messages. Some of the participants especially those from LIG also expressed the need for innovative written material and someone to assist them in using IT tools. These findings reflected the feasibility and acceptability of proposed intervention.

***Discussion***

Effective nutrition education for reducing dietary intake of salt, sugar and fat and improving vegetables and fruit consumption require understanding of influencers of these behaviours, such as, cultural, socio-economic, individual choices etc. All identified themes indicated the need for considering these influencers during intervention development and implementation. Findings of FGDs suggested that intervention should focus on increasing knowledge and awareness about benefits of dietary change, risk perception e.g., hypertension due to high intake of salt, enhancing self-efficacy, time management and involvement of family members. The themes and subthemes helped in deciding on the content of the dietary education material and messages. Based on the suggestions we added some aids for behaviour change, such as, measuring spoons, dining table mats and kitchen calendars besides regular messages.

Use of narrative thematic analysis informed by framework analysis, and techniques from grounded theory approach led to a context-specific intervention. Emergent codes which arose through thematic analysis introduced us to the ground realities of our study setting. There is some evidence on the usefulness of combination of these analysis approaches for smoking cessation and nutrition education intervention in rural South India where the intervention was developed based on this analysis. However, there was need for testing it in urban Indian setting of diverse population.

Qualitative methods and purposive sampling has its limitations of generalizability. However, the purpose of FGDs was to explore the issues related to behaviour change at individual as well as family level so as to come up with acceptable and implementable intervention. Qualitative formative research, despite limitations, led us to the development of a more feasible, culturally acceptable intervention behaviour change.

- Knowledge/awareness
- Attitude
- Self-efficacy
- Perceived seriousness
- Perceived susceptibility
- Perceived benefits and barriers
- Perceived pros and cones
- Modifying factors
- No restriction on fat and sugar intake
- Food preferences of family members
- Support from family members
- Availability of healthy food at home
- Social norms
- Religious norms
- Festivals, customs and traditions
- Media influence
- Need for restrictive and supportive policies
- Availability of healthy food in the area
- Accessibility to markets
- Season
- Time
- Individual dietary change
- Family dietary change
- Dietary change at both individual and family level

**Themes**

**Codes**

**Categories**

**Sub-Themes**

- Cognitive factors
- Risk perception
- Perceived benefits and costs
- Personal factors
- Family norms
- Family preferences
- Support from others
- Home environment
- Societal norms
- Culture
- Influence of media
- Policies
- Availability and accessibility
- Contextual factors
- Individual attributes
- Moderating attributes
- Interpersonal attributes
- Socio-cultural and structural attributes
- Contextual attributes

**Figure 3: Thematic map on dietary behaviour**
